# LINC00659 exacerbates endothelial progenitor cell dysfunction in deep vein thrombosis of the lower extremities by activating DNMT3A-mediated FGF1 promoter methylation

**DOI:** 10.1186/s12959-023-00462-x

**Published:** 2023-03-08

**Authors:** Bo Zhang, Jie Qin

**Affiliations:** 1 Department of Peripheral Vessel, The First Affiliated Hospital of Xi’an Jiaotong University, Xi’an, 710061 Shanxi China; 2 Department of Gastroenterology, The First Affiliated Hospital of Xi’an Medical University, Xi’an, 710061 Shanxi China

**Keywords:** Lower extremity deep vein thrombosis, LINC00659, EIF4A3, FGF1, DNMT3A

## Abstract

It has been shown that long non-coding RNA (lncRNA) LINC00659 was markedly upregulated in the peripheral blood of patients with deep venous thrombosis (DVT). However, the function of LINC00659 in lower extremity DVT (LEDVT) remains to be largely unrevealed. A total of 30 inferior vena cava (IVC) tissue samples and peripheral blood (60 ml per subject) were obtained from LEDVT patients (*n* = 15) and healthy donors (*n* = 15), and then LINC00659 expression was detected by RT-qPCR. The results displayed that LINC00659 is upregulated in IVC tissues and isolated endothelial group cells (EPCs) of patients with LEDVT. LINC00659 knock-down promoted the proliferation, migration, and angiogenesis ability of EPCs, while an pcDNA-eukaryotic translation initiation factor 4A3 (EIF4A3), a EIF4A3 overexpression vector, or fibroblast growth factor 1 (FGF1) small interfering RNA (siRNA) combined with LINC00659 siRNA could not enhance this effect. Mechanistically, LINC00659 bound with EIF4A3 promoter to upregulated EIF4A3 expression. Besides, EIF4A3 could facilitate FGF1 methylation and its downregulated expression by recruiting DNA methyltransferases 3A (DNMT3A) to the FGF1 promoter region. Additionally, LINC00659 inhibition could alleviate LEDVT in mice. In summary, the data indicated the roles of LINC00659 in the pathogenesis of LEDVT, and the LINC00659/EIF4A3/FGF1 axis could be a novel therapeutic target for the treatment of LEDVT.

## Introduction

Venous thromboembolism is a common vascular disease in clinic, including deep venous thrombosis (DVT) and pulmonary embolism (PE). DVT refers to thrombosis that occurs in deep veins, including femoral, popliteal, and intermuscular veins, and it can be divided into upper and lower extremities according to the site of occurrence, in which DVT is more common in the lower extremities [1]. When a DVT patient's embolus dislodges later and subsequently circulates into the lung, it can lead to PE, seriously threatening the patient's life [2]. The typical clinical features of patients with lower-extremity deep venous thrombosis (LEDVT) are muscle soreness and lower extremity swelling or tenderness, but specificity is lacking. Nearly half of LEDVT patients present without typical clinical manifestations, which can easily cause missed diagnosis and misdiagnosis, thus increasing the difficulty of diagnosing and diagnosing LEDVT. In addition, 20%—50% of patients with DVT will develop deep vein post-thrombotic syndrome even if properly treated, which can present with intractable edema, pain, heaviness, hyperpigmentation, and even venous ulceration [3]. The development of post-thrombotic syndrome is also associated with an increased risk of LEDVT recurrence, severely affecting patients' quality of life and increasing the economic burden on families and society [4, 5]. Therefore, timely, and accurate diagnosis of LEDVT and adopting effective interventions are of great importance to alleviate patients' suffering and even save patients' lives.

At present, with the in-depth understanding of endothelial progenitor cells (EPCs) and the increase of research, it has become the focus of medical research [6]. An increasing number of studies have demonstrated that EPCs are of great value in regenerative medicine and clinical applications, which can not only directly participate in angiogenesis but also participate in vascularization and endothelial repair by differentiating into endothelial cells [7]. In addition, EPCs can also play a crucial role in neovascularization, blood vessel and tissue repair, and so on, by secreting a variety of cytokines and angiogenic growth factors [8]. EPCs have important potential applications for the clinical treatment of vascular diseases, especially LEDVT [9]. Bone marrow-derived EPCs first enter the microcirculation, which is more abundant in veins and can repair damaged or missing endothelium, promote angiogenesis, and then favor venous vascular remodeling at LEDVT [10]. Therefore, EPCs may serve as potential seed cells for new approaches to biological treatment of LEDVT. However, the number of EPCs in the body and the migration ability to the thrombus site are limited and vulnerable to the in vivo microenvironment, such as high glucose, advanced age, etc., which are the key issues that hinder the clinical application of EPCs [11]. Thus, it is critical to find ways to enhance the function of EPCs.

In recent years, studies have confirmed that long non coding RNAs (lncRNAs) are key molecules in regulating the occurrence and development of vascular diseases and play irreplaceable roles in angiogenesis. Currently, with the development of next-generation sequencing technology, lncRNAs are being discovered in increasing numbers, and the functional roles and mechanisms that they play are also being studied in depth. Many studies have discovered that lncRNAs play an important role in regulating the proliferation or/and migration of EPCs and angiogenesis, which may be conducive to the mechanistic recanalization of LEDVT. For example, LncRNA GUSBP5-AS was able to alleviate thrombus formation by encouraging EPC migration and angiogenesis [12]. Besides, a research has revealed that lncRNA MALAT1 level was increased in DVT tissues [13]. LINC00659 was located in human chromosome 20q13.33. A study compared the lncRNA expression profiles in the peripheral blood of human patients with DVT at high altitude with those of control subjects at high altitude using RNA sequencing and found that LINC00659 was prominently increased in the peripheral blood of patients with DVT at high altitude [14]. However, there is no report on the effect of LINC00659 on EPC migration and angiogenesis and its therapeutic effect and mechanism on LEDVT.

Based on the above reports, we speculated that LINC00659 might participate in LEDVT progression by modulating cell migration and angiogenesis of EPCs. Therefore, in the present study, we examined the regulation of LINC00659 on the biological behaviors of EPCs, such as proliferation, migration, and angiogenesis, among others. We also explored the regulatory correlation between LINC00659 and potential target genes to decode the molecular mechanism of LEDVT regulation by LINC00659.

## Materials and methods

### Clinical specimens

From September 2018 to July 2020, 15 patients with LEDVT and 15 healthy subjects (aged 40–65 years) diagnosed in the First Affiliated Hospital of Xi'an Jiaotong University (Xi’an, China) were included in this study, and inferior vena cava tissue samples and peripheral blood (60 ml per subject) were collected. For the 15 patients with LEDVT, all were obtained during inferior vena cava resection surgery. 4 of these patients underwent surgical resection of the inferior vena cava with common iliac vein, while internal and external iliac vein anastomoses were performed so that blood in the lower extremities was drained through the internal iliac vein compensatory pathway, and 11 patients underwent partial resection of the inferior vena cava with artificial vessel grafting, all of whom recovered well after surgery with normal renal function and blood flow, and no lower extremity edema and other complications. Individuals with hypertension, diabetes, cardiovascular disease, liver and kidney dysfunction or severe infection, and those with diseases such as immune system diseases or active bleeding were excluded. For the 15 healthy subjects, all were obtained during trauma resection surgery of the inferior vena cava, these donors were traumatized resulting in vena cava spasm with endovascular or full-thickness vessel wall injury, which in turn resected part of the inferior vena cava and reconstructed using artificial blood vessels. This study was conducted in accordance with the declaration of Helsinki and approved by the review board of the First Affiliated Hospital of Xi'an Jiaotong University. Each patient gave written informed consent.

### Isolation and cultivation of EPCs

Endothelial progenitor cell isolation, culture methods and characteristics were as described in previous studies. Briefly, peripheral blood mononuclear cells were isolated using Ficoll-Isopaque Plus lymphocyte separation solution (Histopaque-1077, sigma, USA) density gradient centrifugation according to the manufacturer's requirements. Continue incubation of cells in EBM-2 medium (Lonza, Switzerland) supplemented with FBS, place in 37℃, 5% CO_2_ incubator. Around 2 weeks, the cells are seen to form a pebble like colony. Subsequently, cell surface markers including CD34 and CD133 were analyzed by flow cytometry as well as Dil-Ac-LDL/FITC-UAE-1 double immunofluorescence to identify whether EPCs had typical cell surface markers. The process of EPCs used in this experiment was passage 3 to 5 cells.

EPCs were incubated with Dil-Ac-LDL for 12 h at 37° C atmosphere according to the manufacturer's requirements to sufficiently absorb Dil-Ac-LDL and analyze binding with FITC-UEA-1. Or EPCs were cultured with primary antibodies CD133 and CD34. Then, the above treated EPCs will further proceed under paraformaldehyde for 20 min. Representative micrographs were obtained with a microscope (Olympus, Tokyo, Japan).

### Bioinformatics analysis

Microarray data are available with the accession numbers GSE51260 from Gene Expression Omnibus database (GEO, https://www.ncbi.nlm.nih.gov/geo/). The datasets GSE51260 was based on the platform GPL15314, including 6 inferior vena cava tissue samples from LEDVT and 6 from control samples. The difference analysis was performed using the limma package based on the R software, which employs the classical bayson's t-test analysis method with filtering criteria: |log Fold Change|≥ 0.5, adjust *P* < 0.05. Targeted visualization of differential sites. The volcano plot was drawn by using the R language ggplot2 package to demonstrate the differentially expressed genes.

### Animals and experimental groups

A total of 40 specific pathogen-free (SPF) mice were provided by the laboratory animal center of Xi’an Jiaotong University (Xi’an, China), and the study of the experimental animals conformed to the regulations related to animal management. mice were randomly assigned into a sham group (normal mice, *n* = 10), LEDVT group (model mice, *n* = 10), LEDVT + NC shRNA group (model mice, 200 µL of NC shRNA, injected through the anterior inferior vena cava before modeling, *n* = 10) and LEDVT + LINC00659 shRNA group (model mice, 200 µL of LINC00659 shRNA, injected through the anterior IVC before modeling, *n* = 10). Sham group: Sham group: after 10 mice inhaled anesthesia, a 1.5–2.0 cm incision was made in the right middle of the abdomen, the epidermis, subcutaneous tissue, abdominal muscle, peritoneal cavity were sequentially opened, and the intestinal tube and omentum were separated and covered with warm saline gauze, after which the inferior vena cava was retroperitoneally exposed, the abdomen was closed sequentially with 5–0 prolene (vascular sutures) again. The mice were detected for signs of life, warm adapted, and routinely housed 2 h after the end of surgery. LEDVT group: The contents of the abdominal cavity of ten mice were stripped to the outside of the abdomen, and the steps were the same before. The inferior vena cava was exposed, and the inferior vena cava was divided immediately at the junction between the left renal vein and the inferior vena cava, and then the measured diameter was measured using professional calipers in the simulation of the tied inferior vena cava to establish the model. Next, a 30-G metal needle was placed into the vena cava in parallel with the erector axis of the inferior vena cava using a 7–0 thread at the junction of the left renal vein and inferior vena cava through the back-up, and the metal needle and inferior vena cava were subsequently tied. After the knot was tied, a metal needle was removed from the vertical axis of the inferior vena cava, the head, to prevent damage to the inferior vena cava wall. The protocol of this study was approved by the animal care and use Committee of the The First Affiliated Hospital of Xi’an Jiaotong University.

### Cell lines and culture

Mouse endothelial progenitor cells (EPCs) were obtained from American Type Culture Collection (ATCC, Manassas, VA, USA). The cells were cultured in DMEM medium (ThermoFisher, Shanghai, China) containing 10% fetal bovine serum, 100 U/mL penicillin and 100 μg/mL streptomycin (ThermoFisher, Shanghai, China).

### Cell transfection

The LINC00659 and EIF4A3 full sequence was ligated into pcDNA3.1 plasmid (GenePharma, China). The siRNAs of LINC00659 and FGF1were designed by Qiagen (Frankfurt, Germany) to knock down LINC00659 and FGF1. To avoid off target effects, we designed a total of 4 different siRNAs against LINC00659, si48, si175, si758, and si820. si48: 5’- CGA GGT CTG TAG AGG TGA CAT TGC A-3’; si175: 5’- TCC TGG GCA GCT GAC TTC AAG TTT G-3’. si758: 5’- CAC TCA TGG CGG GTC CAC CTT AAT T-3’; si820: 5’- CAA GAG GCA ACT GTC TGT TTC TAT A-3’. We selected the most efficient siRNA (si758) for subsequent studies. Besides, we selected the 2 best performing siRNA (si758 and si175) constructs into the pLKO.1 vector to construct LINC00659 shRNA. NC siRNA: 5’-CATACGTTGTCTAGGAAGCACAACA-3’. we designed a total of 4 different siRNAs against FGF1, si55, si2209, si7353, and si8782. si55: 5’- CAT ACC AGT GTC AGC TGC ACT TGT A-3’; si2209: 5’- CAT TGA CTG TCA GGA TTG CAG TTT A-3’. si7353: 5’- CAG GTT CCT TCT GAA ATG AAC TGT A-3’; si8782: 5’- GAG TGG CAT GCA GCA TCC AAT TTC T-3’. We selected the most efficient siRNA (si8782) for subsequent studies. The cells were observed to grow to about 70% confluence when the cell monolayer was covered with serum-free DMEM medium, plasmid transfection was performed using Lipofectamine®^3000^ (Thermofisher, USA). Forty-eight hours after transfection, cells were obtained for further analysis.

### Quantitative real-time polymerase chain reaction (RT-qPCR)

Cells from each group were collected, Total RNA was obtained using Trizol assay reagent according to the manufacturer's instructions. RNA (1 µg) was used to generate first-strand cDNA. RT-qPCR was performed using cDNA as a template, and the PCR products were detected with the stepone-plus real time PCR system (Thermo Fisher, USA), with three replicate wells set for each group and GAPDH as the internal reference. The relative expression levels were calculated by using 2^−ΔΔCT^ method.

### Western blotting

Total cell protein was extracted with RIPA lysate, and the protein concentration was determined by using a BCA protein assay kit (Thermo Fisher, USA) in a microplate reader. After denaturation for 10 min with the addition of loading buffer, 50 μg of protein samples were subjected to SDS-PAGE and transferred onto PVDF membranes. The membrane was blocked with blocking solution (5% nonfat dry milk) for 2 h, TBST washed three times, specific primary antibodies were added, respectively. Goat anti rabbit IgG (1:3000, Abcam, ab6721) conjugated with horseradish peroxidase (HRP) was incubated at room temperature for 1 h. Secondary antibodies were added after three TBST washes and then incubated on a shaker. After the membrane was cleaned and the protein exposure was performed, Image J software was applied to detect and analyze the gray values of protein bands on the membrane. The following primary antibodies: anti-β-actin (1:1000, Abcam, ab8227), anti-DNMT3A (1:1000, Abcam, ab2850), anti-EIF4A3 (1:2000, Abcam, ab32485), anti-FGF1 (1:1000, Abcam, ab207321). β-actin was used as an endogenous control.

### Cell proliferation assay

Cell Counting Kit-8 (CCK-8) measurements (Sigma-Aldrich, St. Louis, MO, USA) were performed to examine the proliferation of EPCs. 1 × 10^5^/ml cells from each group after transfection were resuspended, and then press 100 μL/well were seeded into 96 well plates. Three replicates/group were set up so that the 96 well plates were routinely incubated inside the oven. After 24 h incubation, 10 µL of CCK-8 solubility product was added per well and incubated with EPCs for an additional at 37 °C and 5% CO_2_ in a humidified atmosphere. And then OD value at 450 nm was measured and the reading was recorded. After that, the cells in each group after 24, 48, 72, and 96 h of culture were measured by the same method, and the values were recorded and growth curves were plotted.

### Transwell assay

Each group of cells in logarithmic growth phase was taken and starved for 24 h. Cells were digested resuspended to a concentration of 1 × 10^5^/ml. 0.2 ml of the cell suspension was added to the upper chamber of the Transwell, and 700 μL of pre chilled DMEM cell culture with 10% FBS was added to the lower chamber. The Transwell chambers were then placed in an incubator containing 5% CO_2_, 37 °C, and the cells on the upper chamber and basement membrane were swabbed after 24 h using a wet cotton swab. Subsequently, methanol was used to fix the cells for 30 min, and 0.1% crystal violet was used to stain the cells for 20 min. It was finally observed and photographed under an inverted microscope (Olympus Corp, Tokyo, Japan). Five fields were randomly selected, and the number of membrane penetrating cells was counted.

### Tube formation assay

Matrige was thawed well in 4° C freezer. Then 50 µl of Matrigel was taken to spread evenly in a 96 well culture plate and avoid air bubbles. The 96 well plates were placed in a 37 °C CO_2_ incubator for 30 min to allow the gels to solidify. EPCs from each group were digested and a cell suspension was made, adjusted to a cell concentration of 2 × l0^5^/ml, and 150 µl of cells was seeded per well. The 96 well plates were placed in a 37 °C, 5% CO_2_ incubator, and after 12 h, photographs were observed under an inverted microscope. Four fields were randomly patted per well and the number and length of tube formation per field were counted using ImageJ software.

### Chromatin immunoprecipitation (ChIP)

275 µl of 37% formaldehyde was added to 10 ml of medium and incubated for 10 min. Cocktail II and 1 ml of 10 × glycine were subsequently added in cells. SDS was used to resuspend the cells to obtain cell lysates, and 100 µl of the sample was added to 900 µl of a dilution containing cocktail II and 60 µl of protein G agarose. Target antibody and negative control antibody IgG were added to the supernatant for immunoprecipitation with DNMT3A antibody. The collected DNA was directly subjected to RT-PCR, and the amplified products were used for agarose gel electrophoresis. The FGF1 forward primer: 5’- AAG CTG CAG CCA TGA TGG AA -3’, Reverse primer: 5’- CTC CAG GAG CGG GAG GTG -3’.

### Co-immunoprecipitation (Co-IP)

400 µl of PBS (Thermo Fisher, USA) were added to the centrifuge tube, and then the magnetic beads (Takara Biotechnology, Dalian, China) were vortexed for 4 min to mix thoroughly. The supernatant was discarded after magnetic separation, and then 400 μl of PBS was added to resuspend the magnetic beads for use. 500 μl of PBS was added to each tube along with EIF4A3 or DNMT3A antibody at a final concentration of 1 μg/ml. Magnetic separation was followed, in brief, washing the surface unbound antibodies by gently pipetting the magnetic beads with 400 µl of PBS. After washing 2 times magnetically separated and the supernatant was discarded. 500 μl of 0.5% BSA blocking solution was then added and blocking was performed on a rotating shaker at room temperature for 1.5 h. Finally, protein samples were collected and stored at—20 ℃.

### Methylation-specific PCR

Cellular DNA from each group was extracted separately for bisulfite modification, and the modified DNA was amplified separately with methylated primer pairs (biometra, Dublin, Ireland). The primers specific for the methylated FGF1 gene promoter were 5’ -AGC CCT GGT GTG GAG CTA TA -3’ and 5’ -GGT CTC GCC ATC TTT GGG AA -3’. The methylation reaction conditions were pre-denaturation at 94° C for 4 min, followed by 35 cycles of denaturation at 94° C for 30 s, annealing at 56° C for 30 s, and extension at 72° C for 30 s, with a final extension at 72° C for 5 min. PCR products were pooled from each group for 5 μl was electrophoresed on a 1% agarose gel to visualize FGF1 methylation.

### RNA Immunoprecipitation (RIP)

Protease inhibitors, EDTA as well as RNase inhibitors (Qiagen, Hilden, Germany) were added to IP lysis buffer to lyse the cells. After the addition of magnetic beads to preclear for 30 min, IgG antibody and EIF4A3 antibody (Abcam, USA) were added, respectively, and then the addition of magnetic beads was rotated to mix for 2 h at room temperature. Aspirate off the supernatant on a magnetic stand and wash the beads with IP lysis buffer. After proteinase K was added to remove the proteins, Trizol was added to extract the RNA and finally subjected to subsequent analysis.

### Luciferase reporter gene assay

EIF4A3 promoter-luciferase reporter vector was generated by subcloning the PCR amplification product of the EIF4A3 promoter into the upstream of the pGL3-Basic vector (Promega, USA). EPCs were co-transfected with the above plasmids together with pcDNA-3.1 or pcDNA-LINC00659 plasmids. After 48 h, luciferase activity was detected by utilizing the luciferase reporter gene assay kit (Promega, USA). The luciferase activity was calculated with the normalization of Renilla luciferase.

### Immunofluorescence

The transfected EPCs were placed on a glass cover for 24 h. The cells were washed with PBS and fixed in 4% paraformaldehyde for 10 min at room temperature. After 3 washes, cells were placed at room temperature and blocked with immunostaining blocking buffer for 1 h. Immunostaining was performed with TRITC Phalloidin (CA1610, Solarbio) to determine F-actin expression. Nuclei were stained with DAPI. The cover glass was washed with PBS, flip-mounted on glass, and sealed with Fluoromount-GTM. Under the same conditions, images were obtained using inverted fluorescence microscope (XSP-8CA, Shanghai Optical Instrument Factory, China).

### Hematoxylin and eosin (HE) staining

Mouse thrombi containing IVC were stained with HE dye solution according to the manufacturer's instructions. Thereafter, the sections were observed under an inverted microscope (XSP-8CA, Shanghai Optical Instrument Factory, China).

### Statistical analysis

SPSS 22.0 and graphpad prism 7.0 were applied for data analysis and mapping. Student's t-test was performed for comparison of two sample means, and analysis of variance (ANOVA) was used for comparison of means of multiple groups, with *P* < 0.05 considered significant.

## Results

### LINC00659 is upregulated in IVC tissues and peripheral blood of patients with LEDVT

**Fig. 1:**
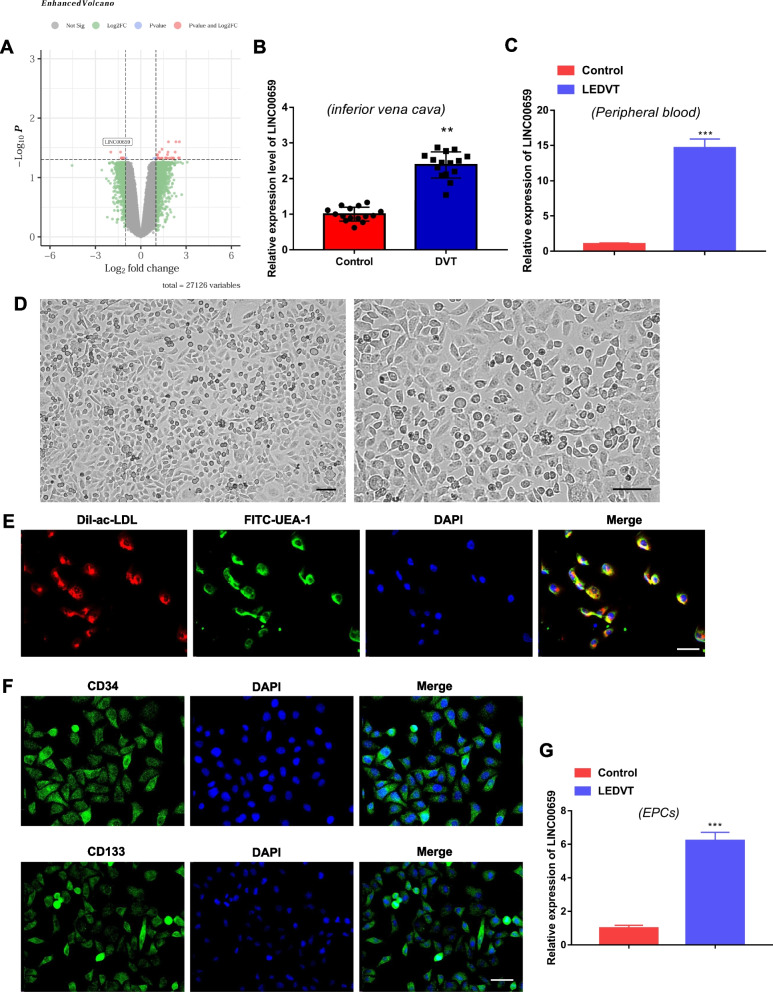
LINC00659 is upregulated in IVC tissues and peripheral blood of patients with LEDVT **A** Volcano plot of differentially expressed lncRNAs in 6 human varicose vein tissues and 6 normal adjacent vein tissues in GSE51260 dataset. **B** RT-qPCR was applied to check LINC00659 level in the IVC tissues of patients with LEDVT (*n* = 15) and healthy controls (*n* = 15). **C** RT-qPCR detection of LINC00659 level in peripheral blood of patients with LEDVT (*n* = 15) and healthy controls (*n* = 15). **D** EPCs showed a cobblestone like morphology on day 3 after the first passage (*n* = 6), Scale bar = 25 μm. **E** Dual fluorescence labeling method of Dil-Ac-LDL/FITC-UAE-1 evaluation of the physiological behaviors of the EPCs (*n* = 4), Scale bar = 25 μm. **F** Immunofluorescence evaluation of CD34 and CD133 expression in EPCs (*n* = 4), Scale bar = 25 μm. G: RT-qPCR detection of LINC00659 level in EPCs isolated from patients with LEDVT and healthy controls (*n* = 6). Each experiment was repeated for 3 times. **P < 0.01, **P < 0.01 *vs* Control group

It is well known that LEDVT is one of the many serious complications of varices [15]. LINC00659 was aberrantly expressed in the venous tissues of patients with varicose veins by GSE51260 dataset microarray analysis (Fig. 1A). According to this, we tried to explore the expression profile of LINC00659 in LEDVT. A total of 30 IVC tissues and peripheral blood were collected for this study, including 15 LEDVT patients and 15 healthy controls. As shown in Fig. 1B and C, LINC00659 level was prominently increased in the IVC tissues and peripheral blood from LEDVT patients compared to the controls. Subsequently, we aimed to further analyze whether the expression of LINC00659 was similarly dysregulated in LEDVT derived EPCs. EPCs from peripheral blood of LEDVT patients and healthy subjects were isolated by density gradient centrifugation. Cells were found to grow in cobblestone assembly after subculture and grew rapidly to approximately 80–90% confluence (Fig. 1D). It was subsequently confirmed by flow cytometry that EPCs mainly expressed the antigen markers CD133 and CD34 (Fig. 1E). We further used the dual fluorescence labeling method of Dil-Ac-LDL/FITC-UAE-1 to evaluate the physiological behaviors of the cells, and the results displayed that the isolated cells were positive for dual fluorescence staining of Dil-Ac-LDL/FITC-UAE-1, which further confirmed that the EPCs were successfully cultured (Fig. 1F). Next, RT-qPCR results suggested that LINC00659 expression was dramatically upregulated in EPCs isolated and cultured from peripheral blood of LEDVT patients compared with controls (Fig. 1G).

### LINC00659 regulates the biological behaviors of EPCs

**Fig. 2:**
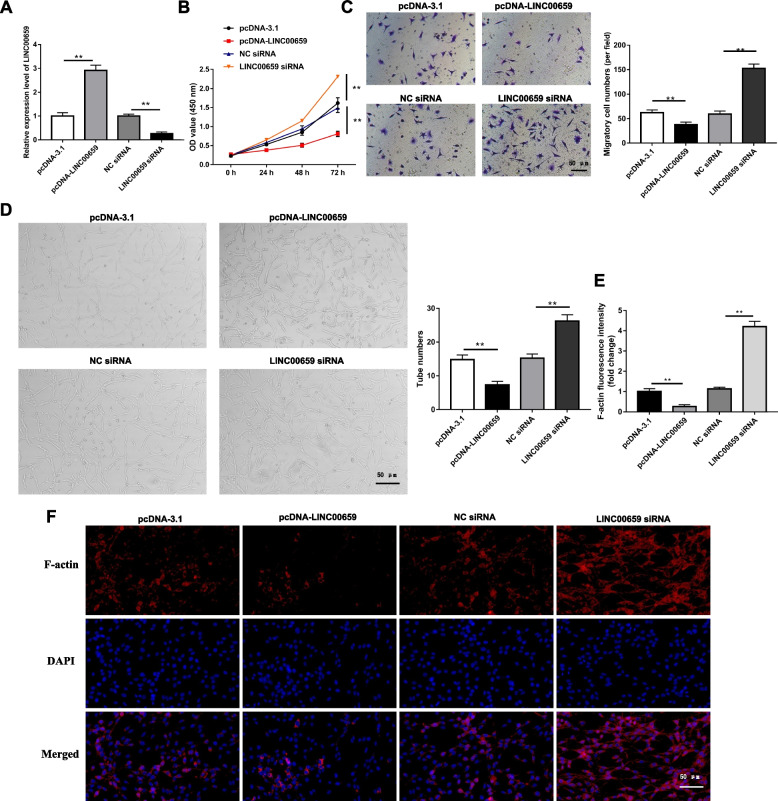
LINC00659 inhibits the proliferation, migration, and angiogenesis ability of EPCs. EPCs were transfected with pcDNA-LINC00659 or LINC00659 siRNA alone for 24 h. **A** RT-qPCR detection of the LINC00659 expression level of EPCs transfected with pcDNA-LINC00659 or LINC00659 siRNA (*n* = 6). **B** EPC, transfected with pcDNA-LINC00659 or LINC00659 siRNA, proliferation was checked by CCK-8 assay (*n* = 6). **C** Transwell assay was applied to test the effects of pcDNA-LINC00659 or LINC00659 siRNA transfected on EPC migration (*n* = 6). **D** Tube formation assay was applied to test the effects of pcDNA-LINC00659 or LINC00659 siRNA transfected on EPC angiogenesis (*n* = 6). **E**–**F** Effect of pcDNA-LINC00659 or LINC00659 siRNA transfected on actin cytoskeleton structure in cultured EPCs (*n* = 4). Each experiment was repeated for 3 times. **P < 0.01

To further explore the effect of LINC00659 on endothelial progenitor cell proliferation, migration, and angiogenesis. EPCs were transfected with pcDNA-LINC00659 or LINC00659 siRNA alone for 24 h. The efficiency of pcDNA-LINC00659 and LINC00659 siRNA on its level in EPCs was tested by RT-qPCR (Fig. 2A). As shown in Fig. 2B, upregulation of LINC00659 could reduce the proliferative capacity of EPCs as compared to the pcDNA-3.1. Moreover, the proliferative capacity of EPCs was strengthened in the LINC00659 siRNA group. In addition, the results of EPC migration ability detected by Transwell assay displayed that LINC00659 overexpression substantially suppressed cell migration, whereas LINC00659 knockdown significantly encouraged cell migration ability (Fig. 2C). Studies have found that angiogenesis of EPCs plays a critical role in thrombus recanalization. To investigate the regulatory effect of LINC00659 on EPC angiogenesis, and to determine the microvascular morphology of endothelial cells by in vitro tube formation method. As shown in Fig. 2D, both tube number and length of EPCs transfected with pcDNA-LINC00659 were memorably inhibited compared to pcDNA-3.1, indicating a negative effect of LINC00659 on EPC angiogenesis. Similarly, the tube formation ability of EPCs was enhanced after LINC00659 knockdown. In this paper, the link between LINC00659 and cytoskeleton in cells was also discussed in depth. As can be seen from Fig. 2E and F, LINC00659 impaired the expression of F-actin, while LINC00659 knock-down inhibited the structure of F-actin. It is suggested that LINC00659 plays an important role in regulating the proliferation, migration, and angiogenesis of EPCs in vitro, and it can at least partially regulate the expression of F-actin.

### LINC00659 positively regulated EIF4A3 expression

**Fig. 3:**
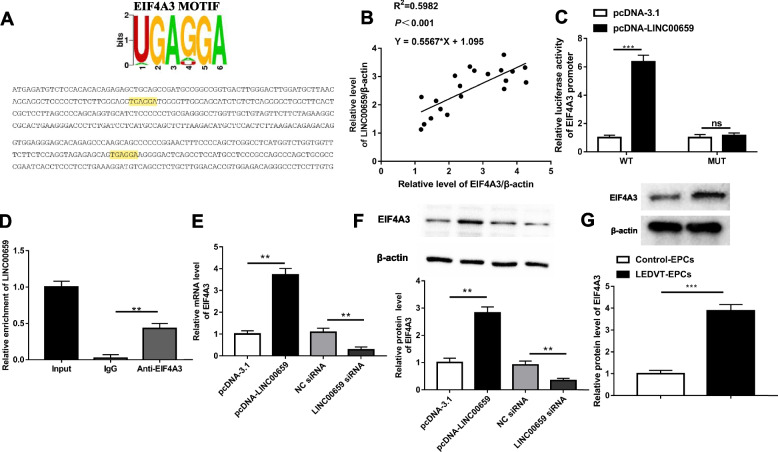
LINC00659 upregulated EIF4A3 expression. **A** The consensus sequence of EIF4A3 motif, and the possible binding sites of LINC00659 and EIF4A3. **B** The relationship between LINC00659 and EIF4A3 in EPCs (*n* = 6). **C** Luciferase reporter assay investigated whether LINC00659 could bind to the EIF4A3 promoter at the putative sites (*n* = 6). **D** The enrichment of LINC00659 in the EIF4A3 precipitate was determined by RIP (*n *= 6). **E** RT-qPCR displayed the EIF4A3 mRNA expression with pcDNA-LINC00659 or LINC00659 siRNA transfection (*n* = 6). **F** Western blotting displayed the EIF4A3 protein level with pcDNA-LINC00659 or LINC00659 siRNA transfection (*n* = 6). **G** Western blotting detection of EIF4A3 level in EPCs isolated from patients with LEDVT and healthy controls (*n* = 6). Each experiment was repeated for 3 times. ***P < 0.001, **P < 0.01.

Next, we analyzed the possible target genes of LINC00659 by using an online biological website (http://starbase.sysu.edu.cn/). Figure 3A displayed that there was a binding sequence for LINC00659 on the Eukaryotic initiation factor 4A-III (EIF4A3) promoter. We further validated the expression correlation between them by Pearson correlation test, and the results suggested that LINC00659 and EIF4A3 level exhibited significant positive correlation in EPCs (Fig. 3B). Subsequently, to evaluate whether LINC00659 directly binds to the promoter region of the EIF4A3 gene, a promoter sequence containing EIF4A3 binding sites was cloned into the upstream luciferase gene of the pGL3-basic-Luc vector, and a luciferase plasmid containing EIF4A3 promoter mutations was constructed. The results showed that the luciferase activity containing the EIF4A3 promoter sequence was significantly increased after LINC00659 overexpression, whereas the luciferase activity was abolished after the binding site was mutated (Fig. 3C). Besides, the results of RIP assay further suggested that LINC00659 RNA level was markedly enriched in EIF4A3 precipitate, confirming the association relationship (Fig. 3D). Additionally, the mRNA and protein levels of EIF4A3 in EPCs were memorably upregulated after LINC00659 overexpression, whereas LINC00659 inhibition could downregulated EIF4A3 expression (Fig. 3E-F). Interestingly, Western blotting results also revealed that EIF4A3 expression was dramatically upregulated in EPCs isolated and cultured from peripheral blood of LEDVT patients compared with controls (Fig. 3G). Taken together, these results concluded that LINC00659 was able to promote EIF4A3 expression.

### EIF4A3 overexpression could reverse the facilitative effect of LINC00659 interference on EPCs migration and angiogenesis.

**Fig. 4:**
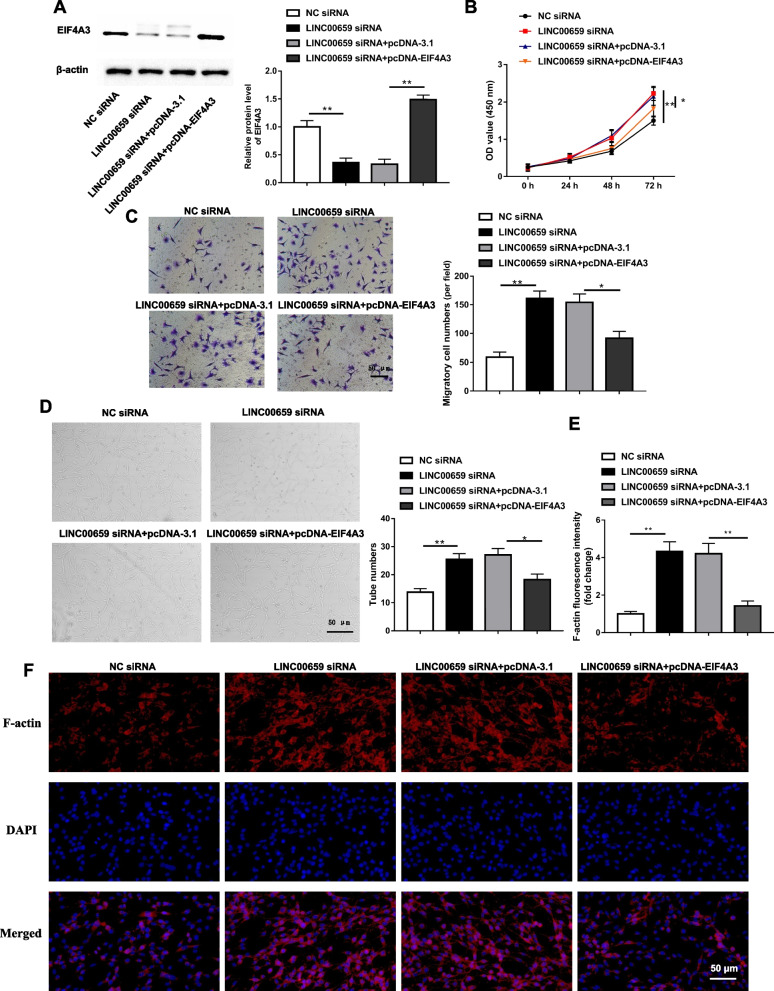
EIF4A3 overexpression reversed the promoting effect of LINC00659 interference on EPCs migration and angiogenesis. LINC00659 siRNA was transfected alone or together with pcDNA-EIF4A3 into EPCs for 24 h. **A** Western blotting was employed to determine the levels of EIF4A3 in EPCs transfected with LINC00659 siRNA or/and pcDNA-EIF4A3 (*n* = 6). **B** The cell proliferation of EPCs transfected with LINC00659 siRNA or/and pcDNA-EIF4A3 was checked by CCK-8 assay (*n* = 6). **C **Transwell detection of the cell migration of EPCs transfected with LINC00659 siRNA or/and pcDNA-EIF4A3 (*n* = 6). **D **Tube formation assay was applied to test the effects of LINC00659 siRNA or/and pcDNA-EIF4A3 on EPC angiogenesis (*n* = 6). **E**–**F** Effect of LINC00659 siRNA or/and pcDNA-EIF4A3 on actin cytoskeleton structure in cultured EPCs (*n* = 4). Each experiment was repeated for 3 times. *P < 0.05, **P < 0.01

To determine whether LINC00659 was involved in the regulation of EPCs biological function by activating EIF4A3 expression, rescue experiments were applied with co-transfection of pcDNA-EIF4A3 with LINC00659 siRNA plasmid. Figure 4A is shown as the effect of pcDNA-EIF4A3 in EPCs on EIF4A3 level. Subsequently, with CCK-8, Transwell, Tube formation and immunofluorescence assays, EIF4A3 overexpression was able to partially reverse the promoting effects of LINC00659 interference on EPCs proliferation (Fig. 4B), migration (Fig. 4C), angiogenesis (Fig. 4D), and the formation of F-actin cytoskeletal structures (Fig. 4E-F).

### EIF4A3 recruits DNMT3A to the FGF1 promoter region and represses FGF1 expression

**Fig. 5:**
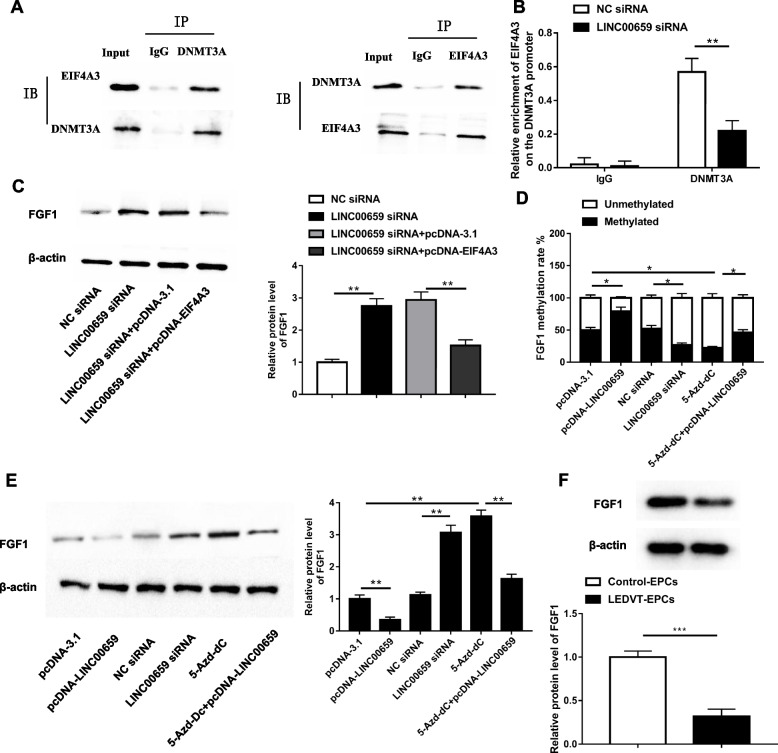
EIF4A3 recruits DNMT3A to the FGF1 promoter region and represses FGF1 expression. **A** Co-IP was employed to check the interaction between EIF4A3 and DNMT3A (*n* = 6). **B** The enrichment of DNMT3A in the FGF1 promoter region was tested by ChIP assay (*n* = 6). **C** Western blotting showed the FGF1 protein expression with LINC00659 siRNA or/and pcDNA-EIF4A3 transfection (*n* = 6). **D **Methylation levels of the FGF1 promoter in EPCs were checked by MSP-PCR (*n* = 6). **E** Western blotting was applied to test the protein level of FGF1 in EPCs (*n* = 6). **F** Western blotting detection of FGF1 level in EPCs isolated from patients with LEDVT and healthy controls (*n* = 6). SGI-1027: DNMT3A inhibitor (8 µM). Each experiment was repeated for 3 times. *P < 0.05, **P < 0.01, ***P < 0.001

With the help of online bioinformatics tools, we discovered that methyltransferases 3A (DNMT3A) might serve as an EIF4A3 target. To verify that DNMT3A is an interacting protein of EIF4A3, we performed Co-IP experiments. The results displayed a large amount of DNMT3A protein blotted in the EIF4A3 precipitates, and similarly, a large amount of EIF4A3 protein was also found in the DNMT3A immunoprecipitates (Fig. 5A). The existence of a regulatory role for FGF1 in LEDVT was previously established in our laboratory. Next, we sought to hypothesize whether there is an association of FGF1 with DNMT3A. Interestingly, ChIP assay experiments found that DNMT3A was significantly enriched in the FGF1 promoter region, which was prominently prevented by LINC00659 knockdown (Fig. 5B). We also found that FGF1 protein level was obviously increased after LINC00659 downregulation, which was partially counteracted by EIF4A3 overexpression (Fig. 5C). Moreover, LINC00659 upregulated increased FGF1 promoter methylation and suppressed FGF1 protein level, whereas LINC00659 knockdown downregulated FGF1 promoter methylation and encouraged FGF1 level. Besides, overexpression of LINC00659 dramatically inversed the repression effect of DNMT3A inhibitor 5-Aza-Dc on FGF1 promoter methylation, and the facilitative effect on FGF1 expression (Fig. 5D and E). We also displayed that FGF1 protein level was substantially upregulated in EPCs isolated and cultured from peripheral blood of LEDVT patients compared with controls (Fig. 5F). Taken together, EIF4A3 facilitated DNMT3A-mediated FGF1 promoter methylation.

### Interference with FGF1 reversed the promoting effect of LINC00659 inhibition on endothelial progenitor cell migration and angiogenesis

**Fig. 6:**
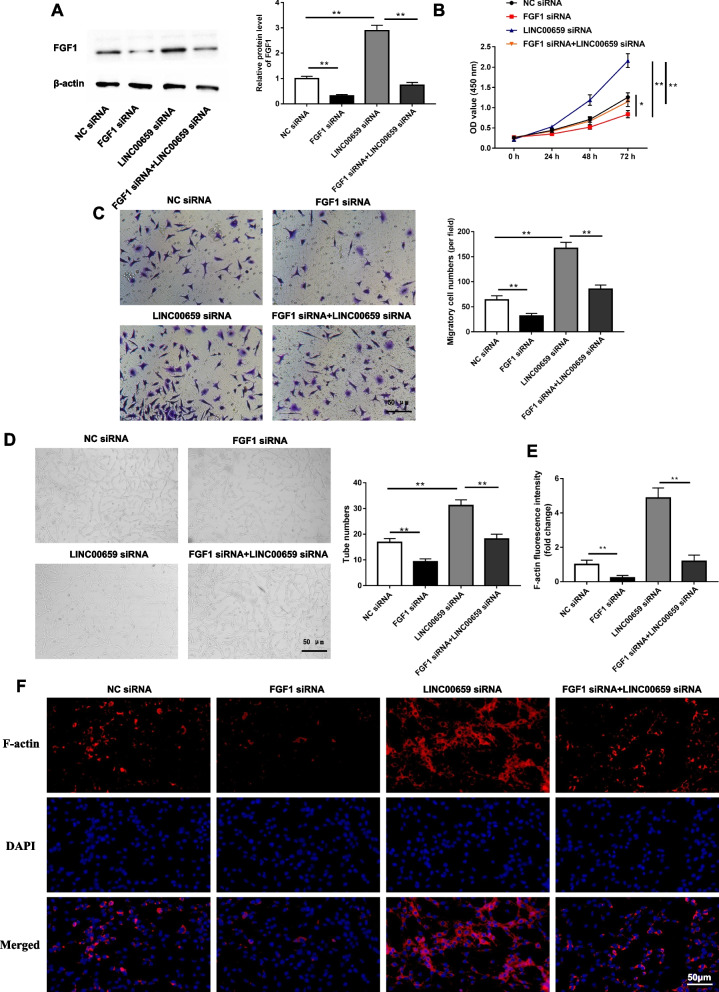
Interference with FGF1 reversed the facilitative effect of LINC00659 inhibition on endothelial progenitor cell migration and angiogenesis. LINC00659 siRNA and FGF1 siRNA were transfected individually or together into EPCs. **A** Western blotting was applied to determine the levels of EIF4A3 in EPCs transfected with FGF1 siRNA or/and LINC00659 siRNA (*n *= 6). **B** The cell proliferation of EPCs transfected with FGF1 siRNA or/and LINC00659 siRNA was tested by CCK-8 assay (*n* = 6). **C** Transwell detection of the cell migration of EPCs transfected with FGF1 siRNA or/and LINC00659 siRNA (*n* = 6). **D** Tube formation assay was employed to check the effects of LINC00659 siRNA or/and FGF1 siRNA on EPC angiogenesis (*n* = 6). **E**-**F** Effect of LINC00659 siRNA or/and FGF1 siRNA on actin cytoskeleton structure in cultured EPCs (*n* = 4). Each experiment was repeated for 3 times. **P < 0.01.

Next, according to the relationship between LINC00659 and FGF1, we transfected LINC00659 siRNA and FGF1 siRNA individually or together into EPCs. Figure 6A suggested that FGF1 levels in EPCs were reduced by approximately threefold after transfection of FGF1 siRNA. FGF1 inhibition was revealed to restrain the proliferation (Fig. 6B), migration (Fig. 6C), angiogenesis (Fig. 6D), and formation of F-actin cytoskeletal structures (Fig. 6E-F) of EPCs. Moreover, we observed that the positive effects on EPCs cell behaviors after LINC00659 downregulated could be largely blocked by FGF1 inhibition. In a word, our data demonstrated that the suppressive role of LINC00659 in maintaining the behavioral function of EPCs largely depends on the EIF4A3/DNMT3A/FGF1 axis.

### Knockdown of LINC00659 alleviates LEDVT in mice

**Fig. 7:**
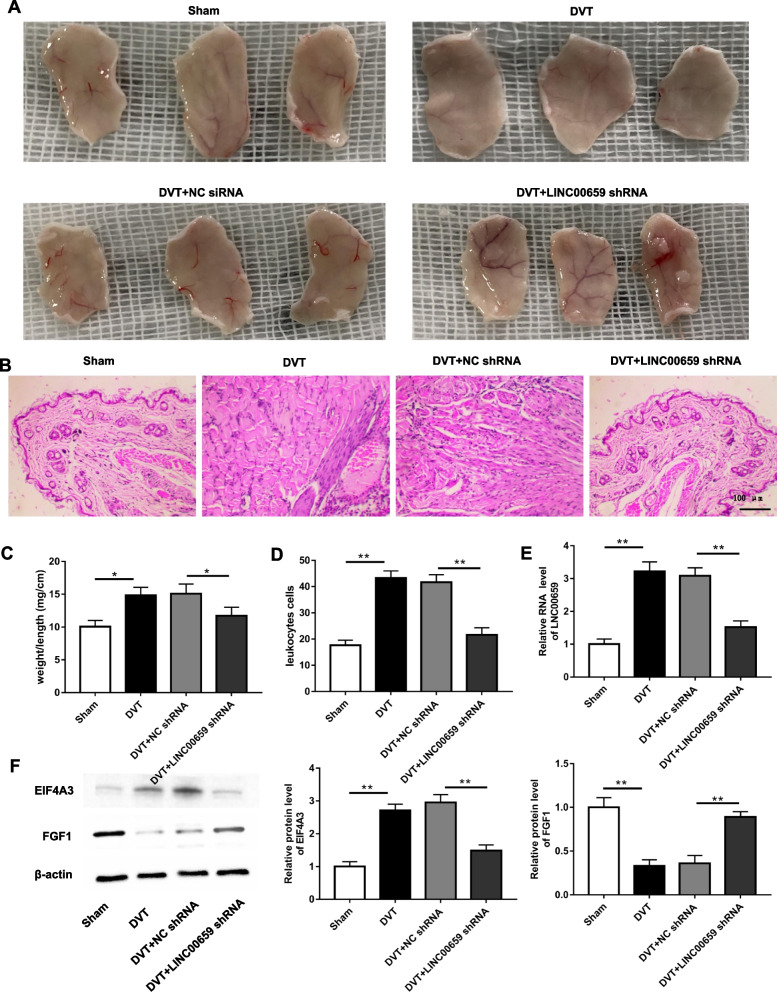
Knockdown of LINC00659 alleviates LEDVT in mice. **A** In vivo angiogenesis was assessed on day 7 after subcutaneous injection of LINC00659 shRNA lentivirus into mice (*n* = 10). **B** HE staining was applied to check IVC thrombosis pathological changes (*n* = 10). **C** The ratio of thrombus tissue weight to length in mice of each group (*n* = 10). **D** Leukocyte numbers in thrombi of mice in each group. **E **The level of LINC00659 in IVC was checked by RT-qPCR (*n* = 10). **F**–**H** The protein levels of EIF4A3 and FGF1 in IVC were tested by Western blotting (*n* = 10). *P < 0.05, **P < 0.01

To examine the effects of LINC00659 on LEDVT, we performed and injected LINC00659 shRNA lentivirus and observed EPC angiogenesis in vivo. The results of the in vivo angiogenesis assay showed that mice in the LEDVT group had significantly less angiogenesis, whereas the angiogenic capacity was restored after LINC00659 knockdown (Fig. 7A). Further, compared with the sham group, obvious intraluminal thrombosis was observed in LEDVT, with loose cell arrangement and aggravated tissue damage, all of which were above reversed by LINC00659 knockdown (Fig. 7B). However, mice in the LINC00659 shRNA group had a continuous increase in vascular endothelial cells in the IVC, whereas inflammatory cell infiltration, intraluminal thrombus, and collagen fibrotic proliferation were decreased (Fig. 7B). Additionally, in comparison with the sham group, the ratio of thrombus weight to length was decreased significantly in the LINC00659 shRNA group, and the number of inflammatory leukocytes was also substantially reduced in the LINC00659 shRNA group (Fig. 7C-D). Consistent with in vitro findings, compared with the sham group, the LEDVT group showed prominently increased LINC00659 and EIF4A3 expression and downregulated FGF1 expression in the IVC of mice, whereas in the LINC00659 shRNA group, LINC00659 and EIF4A3 expression was decreased and FGF1 protein level was significantly increased (Fig. 7E-F).

## Discussion

Early detection of patients with LEDVT and aggressive management are particularly important to prevent disease progression. Therefore, exploring new biomarkers for the diagnosis of LEDVT is of great importance to avoid or reduce the progress of traumatic examinations, early diagnosis and treatment, and prevention of complications during the patient's treatment [16]. This study isolated EPCs from the peripheral blood of LEDVT patients and healthy subjects by density gradient centrifugation, followed by flow cytometry and confirmed that EPCs mainly expressed the antigen markers CD133 and CD34, and the isolated cells were found to have the physiological behavior of EPCs as assessed by double fluorescence labeling with Dil-Ac-LDL/FITC-UAE-1. At present, EPCs are commonly identified using a combination of several antigens including CD34, CD133, and FLK-1. CD34, a sialomucin with a molecular weight of 110 Ku, is selectively expressed on hematopoietic stem cells and some activated vascular endothelial cells J, where it functions as an adhesion molecule for the interaction between endothelial cells and haematopoietic precursor cells [17]. The exact function of CD133, a cholesterol binding glycoprotein selectively expressed by haematopoietic stem and progenitor cells with a molecular weight of 120 Ku, remains unknown, but cells positive for CD133 can differentiate into a variety of cellular phenotypes, including endothelial cells [18]. Studies have confirmed that either early EPCs or late EPCs are able to bind lectins-Ulex Europaeus Agglutinin I rendering the cells green and able to engulf Dil-labeled acetylated low-density lipoprotein rendering the cells red. Thus, an EPC that is differentiating can be identified when both markers are positive [19]. Further, we discovered that LINC00659 was enhanced in IVC tissues and peripheral blood of LEDVT, and overexpression of LINC00659 was able to inhibit EPC proliferation, migration, and angiogenesis. Besides, we established LEDVT mouse models and demonstrated that LINC00659 might be an endogenous regulator of LEDVT. Inhibition of LINC00659 was able to potentially prevent LEDVT progression.

EPCs play an important role in physiological and pathological angiogenesis in adults, where they are recruited to thrombi and accelerate thrombus organizing recanalization [20]. Recently, as emerging RNA molecules with functional roles, lncRNAs play important regulatory roles in biological behaviors such as cell proliferation and migration, and are of great value in the process of angiogenesis, which may bring dawn to the millennium of tumor angiogenesis and ischemic diseases [7, 21–23]. A research displayed that lncRNA MALAT1 level was enhanced in DVT tissues. MALAT1 overexpression could suppress the proliferation and migration of EPCs in DVT [13]. In addition, lncRNA GUSBP5-AS can accelerate EPC migration and angiogenesis and DVT regression, and is considered a favorable aid for LEDVT treatment [12]. Additionally, LINC00659 regulated the expression of prothrombotic genes via its miRNA response element, which could be established as a biomarker of DVT [14]. Our results displayed that LINC00659 could encourage EPC proliferation, angiogenesis, migration, and F-actin filaments. Knockdown of LINC00659 had the opposite effects. In which F-actin and microtubule systems have been demonstrated to be required for cell adhesion and migration [24]. We also found that LINC00659 was able to promote the expression of EIF4A3.

EIF4A3 is a core component of the exon junction complex (EJC) and plays an important role in RNA metabolism [25]. The EJC can regulate brain development, neuronal growth, and neuronal activity [26]. Several recent studies have found that many lncRNAs are involved in molecular regulation through their interactions with proteins. A study suggested that EIF4A3 was identified as a binding protein of lncRNA H19 by immunoprecipitation of RNA binding protein, and lncRNA H19 bound to EIF4A3 and was able to promote colon cancer cell proliferation and affect the expression of cell cycle regulatory genes at the translational or post-translational level [27]. In addition, EIF4A3 has been reported to be aberrantly expressed and identified as a diagnostic marker for many diseases [28–30]. A study has found that EIF4A3 promoted ox-LDL induced aberrant autophagy in HUVECs and reduces plaque stability in vivo [25]. Consistently, in our study, we found that EIF4A3 overexpression reversed the facilitative effect of LINC00659 interference on EPCs migration and angiogenesis. Besides, we also revealed EIF4A3 recruited DNMT3A to the FGF1 promoter region and represses FGF1 expression.

According to the pre-existing findings, the process of angiogenesis is complex and the factors involved are numerous. For example, hypoxia inducible factor [31], fibroblast growth factor (FGF) [32], endothelial nitric oxide synthase [33], matrix metalloproteinase [34], and EPCs, among others, can affect vascular neogenesis. FGFs are a family of heparin integrating growth factors that consist of 23 members [35]. 18 of them act as ligands and bind to four receptor tyrosine kinases (FGFR-1–4), which in turn regulate cell growth, differentiation, and angiogenesis [36]. FGF1 and FGF2 can induce the proliferation and migration of endothelial cells, and have a strong role in promoting angiogenesis [37]. Mutant FGF1 can produce integrin αVβ3, resulting in inhibition of angiogenesis of Human umbilical endothelial cells [38]. It has been reported that upregulation of FGF1 expression to be able to ameliorate atherosclerosis development [39]. Further, a study has revealed that FGF1 expression was significantly downregulated in DVT rats, and overexpressed FGF1 could promote EPC proliferation, migration, and tube formation, thus dissolving thrombi in the veins of DVT rats [40]. DNA methylation refers to the formation of 5-methylcytosine by the transfer of a methyl group from S-adenosylmethionine to position 5 of a cytosine in a cytosine phosphodiester guanine (CpG) site on a DNA strand under the action of DNA methyltransferases (DNMTs) [41]. Among these, DNMTs can be divided into three classes, de novo methylases (DNMT3A/3B), maintenance methylases (DNMT1), and methylation cooperative enzymes (DNMT2 and DNMT3L) [42]. During cell division, DNMT1 can recognize DNA generated during DNA replication where only the mother strand is methylated and then methylate the CpG sequence on the newly synthesized daughter strand, so it is responsible for the maintenance of the methylation pattern [43]. Whereas DNMT2 and DNMT3L do not have the ability to methylate cytosines and usually only serve as linkers for methylation related proteins [44]. Although also capable of methylating hemi-methylated DNA, DNMT3A and DNMT3B mainly function as de novo methyltransferases to establish DNA methylation patterns during embryogenesis [45]. Moreover, DNMT3A and DNMT3B have distinct DNA methylation target sites depending on cell type and developmental stage [46]. It is well known that the secondary satellite DNA region of the genome consists of 50,000–100,000 DNA repeats in the centromeric region, which are specifically methylated by DNMT3B but not by DNMT3A [47]. On the contrary, the main satellite repeats located in the region around the centromere are preferentially methylated by DNMT3A. A study used the MSRF approach and confirmed a CPG site within the G isoform of the FGF1 gene locus that was preferentially methylated by DNMT3A [48]. This further corroborates our results. EIF4A3 could recruit DNMT3A to the FGF1 promoter region and represses FGF1 expression.

In summary, our study substantiated that LINC00667 was prominently elevated in IVC tissues of patients with LEDVT. Overexpression of LINC00659 inhibited the proliferation, migration, and angiogenesis ability of EPCs by facilitating DNMT3A-mediated FGF1 promoter methylation via promoting EIF4A3 expression. These findings may provide a new molecular target and theoretical basis for LEDVT patient treatment.

## Data Availability

The data used to support the findings of this study are available from the corresponding author upon request.
